# Prevalence and associations of problematic smartphone use with smartphone activities, psychological well-being, and sleep quality in a household survey of Singapore adults

**DOI:** 10.1371/journal.pone.0315364

**Published:** 2024-12-18

**Authors:** Rebecca Hui Shan Ong, Hui Shan Sim, Manfred Max Bergman, Choon How How, Constance Ai Li Png, Chau Sian Lim, Lai Huat Peh, Hong Choon Oh

**Affiliations:** 1 Health Services Research, Changi General Hospital, SingHealth, Singapore, Singapore; 2 Care and Health Integration Department, Changi General Hospital, Singapore, Singapore; 3 Department of Social Sciences, University of Basel, Basel, Switzerland; 4 Family Medicine, Academic Clinical Programme, Duke-NUS, Singapore, Singapore; 5 Clinical Psychology Department, Changi General Hospital, Singapore, Singapore; 6 Psychological Medicine Department, Changi General Hospital, Singapore, Singapore; 7 Centre for Population Health Research and Implementation, Singapore Health Services, Singapore, Singapore; Fundación Universitaria del Área Andina, COLOMBIA

## Abstract

**Introduction:**

Despite the many benefits of smartphones, researchers have raised concerns over problematic smartphone use (PSU) and its negative effects on physical and psychological well-being. Studies examining PSU and its impact among adults remain limited. Hence, we aim to examine the prevalence of PSU among adults in Singapore, and explore its associations with smartphone activities, sleep quality, and psychological well-being, as well as age and gender-related differences in these associations.

**Methods:**

A household survey (n = 1200) was conducted among multi-ethnic Singapore adults aged 21 to 60. The survey employed a proportionate stratified random sampling approach. The Smartphone Addiction Scale–Short Version was used to determine risk of PSU. Adjusted multivariable logistic regressions, age-stratified (21–30, and above 30) analyses and sensitivity analyses were performed.

**Results:**

The survey response rate was 45.7%. PSU prevalence rate was estimated to be 34.0%. Adults at risk were younger (OR = 3.72, *p* < 0.001), had poor sleep quality (OR = 2.94), reported depressive (OR = 2.84, *p* = 0.001) or anxiety symptoms (OR = 2.44, *p* < 0.001), tend to use smartphones for social media (OR = 2.81, *p* = 0.002) or entertainment (OR = 2.72, *p* < 0.001). Protective factors include higher levels of social support (OR = 0.76, *p* = 0.007), using smartphones for calling family (OR = 0.39, *p* = 0.003) and friends (OR = 0.53, *p* = 0.030), and spending four hours or less of smartphone usage duration (OR = 0.40, *p* < 0.001). Sensitivity analyses confirmed these findings. Associations between PSU and poor sleep quality (OR = 3.72, *p* < 0.001), depressive (OR = 3.83, *p* < 0.001), and anxiety symptoms (OR = 2.59, *p* = 0.004) and social media usage (OR = 3.46, *p* < 0.001) were more pronounced in adults over 30. PSU was more prevalent among females in those aged 21–30 (OR = 2.60, *p* = 0.022). Social support appears to be a protective factor for adults over 30 (OR = 0.64, *p* < 0.001) but was not observed in those aged 21–30. Among males, younger age (21–30 years), poor sleep quality, depressive symptoms, and anxiety symptoms, and using social media and entertainment apps were significantly associated with PSU. Females showed similar associations. Social support appears to be a protective factor for females (OR = 0.70, *p* = 0.018), but this association was not observed for males. Shorter smartphone usage times were inversely associated with PSU in both genders.

**Conclusion:**

A substantial proportion of adults exhibited PSU. Findings highlight the differential associations between PSU and psychological well-being, social support, interactions with technology, and sleep quality. These associations are influenced by age which has implications for preventive efforts.

## Introduction

Smartphones are widely used and have revolutionized the way we communicate, work, shop, learn, and interact with others [1, 2]. Despite numerous benefits, research has identified negative consequences related to excessive smartphone use [3–6] and proposed constructs such as "smartphone addiction" and "problematic smartphone use" [7–9]. Excessive smartphone use shares symptomatic similarities with behavioural addictions, including salience, mood changes, tolerance, withdrawal, and conflict [10–12]. No diagnostic criteria for smartphone addiction exist to date, although ongoing efforts are addressing this gap [13]. In this study, we employ Problematic Smartphone Use (PSU) in line with recommendations [14] and is defined as: "An inability to regulate one’s use of the mobile phone, which eventually involves negative consequences in daily life" [15].

Psychosocial factors play an important role in the development of PSU. For instance, emotional dysregulation, and the ’fear of missing out’ (FoMO) have been associated with increased vulnerability to PSU as individuals turn to smartphones to manage their negative emotions [16] or engage excessively online to stay socially connected or updated [17]. Gender differences have also been observed, with studies suggesting that females may be at greater risk of PSU due to higher stress levels, anxiety, and higher engagement with social apps. Research suggests that both process-related (e.g. consumption of news, entertainment) and social-related (e.g. social networking, messaging) smartphone use can contribute to the development of PSU. However, it remains unclear which type of use is most strongly associated with PSU [18–20]. Additionally, researchers have observed that females engage with smartphones more frequently for social purposes and experience higher social stress, which could increase their risk of problematic smartphone behaviour [20, 21]. Given the psychological challenges associated with excessive smartphone use, recent studies have explored the potential role of psychological resilience. Resilience has been shown to mediate or moderate the relationship between poor sleep quality, FoMO, mental health, and stress and PSU, potentially acting as a protective factor against PSU [22–24]. Beyond resilience, individual differences, such as personality traits, also play a significant role in susceptibility to PSU. Several studies have found connections between the Big Five personality traits and PSU. Neuroticism correlates positively with PSU, partly due the need to seek reaffirmation for social acceptance and belonging through social media apps [25, 26]. In contrast, conscientiousness has a negative association with PSU, reflecting findings that links low self-regulation to addictive tendencies [26–28]. Extraversion, however, appears unrelated to PSU [26, 29].

Research on PSU has predominantly focused on children and adolescents [16–18], contending that this demographic is particularly vulnerable to sleep disturbances, social dysfunction, academic underperformance, stress, and mental health symptoms [3–6, 19, 20]. However, researchers have emphasised the importance of including adults in PSU research to gain a more comprehensive understanding of PSU [30]. Studies conducted among adults in Spain, Lebanon, and Germany have reported a PSU prevalence of 20.5%, 20.2%, and 20.6%, respectively [31–33], while studies in Hong Kong [34] and Tianjin [35] reported an elevated 39% and 52%, respectively. Research on adults have identified adverse consequences in adulthood [36], such as anxiety, depression, and lower mental well-being among Hong Kong Chinese adults aged 18 years and above [37]. Similarly, associations were observed between PSU and younger age groups, females, prolonged daily smartphone usage, elevated stress, anxiety, depression, and sleep disturbances among German adults aged 16 through 65 [33]. PSU impact on sleep quality has been associated with bedtime procrastination [38, 39]. Zhang and Wu [38] found that bedtime procrastination mediates the relationship between problematic smartphone use and poor sleep quality. They suggest that high levels of smartphone engagement at bedtime, coupled with the reluctance to disengage from activities on smartphones, lead to delays in sleep onset, ultimately resulting in poorer sleep quality and shorter sleep duration.

Despite advancements in PSU research, studies on adult populations, particularly across different age groups, remain limited. Neugarten’s theory of adult development posits that, throughout the human lifespan, societal expectations shapes individuals’ engagement in activities, behaviours, and milestones that are specific to their age groups [40]. Given the variation in lifestyles, family environment and behaviours across ages and its influence on smartphone usage [41], it is crucial to consider the influence of age on PSU. While prior research have examined age-related variations in line with the ’components model of addictions’ [11], there remains a limited body of literature on age-related variations in the prevalence of PSU or its associated factors within a multi-ethnic adult population.

Our study addresses this gap by exploring PSU among adults in Singapore—a multi-ethnic, urbanised city-state with one of the world’s highest smartphone usage rates, where 97% of the population in 2021 were smartphone users [42]. Specifically, we aim to assess the prevalence of PSU among adults in Singapore, and explore its associations with smartphone activities, sleep quality and psychological well-being, and potential age and gender-related differences in these associations.

## Materials and methods

### Participants

A cross-sectional household survey was conducted from October to November 2022, targeting Singaporean citizens and permanent residents aged 21 through 60 who owned a smartphone and were proficient in English, Mandarin, or Malay. The recruitment period spanned from October 19, 2022, when the first participant was recruited, to November 26, 2022, when the last participant was recruited. The study sample was drawn from the eastern region of Singapore, with an approximate population of 426,000 residents [43].

### Measures

The primary outcome, PSU, was evaluated with the Smartphone Addiction Scale–Short Version (SAS-SV) [44]. Suitable reliability and validity measures were reported on this ten-item self-report scale across diverse adult populations, including different cultural backgrounds [34, 45, 46]. The SAS-SV encompassed five domains: daily-life disturbance, withdrawal, cyberspace-oriented relationships, overuse, and tolerance [44]. Responses are measured on a 1–6 ordinal scale, yielding total scores between 10 and 60. Higher scores indicated increased severity of PSU. In accordance with [44], we adopted at risk of PSU cut-off values of 31 for males and 33 for females.

### Sleep quality and psychological well-being measurements

The 19-item Pittsburgh Sleep Quality Index (PSQI) includes seven domains: subjective sleep quality, sleep latency, sleep duration, habitual sleep efficiency, sleep disturbances, use of sleep medications, and daytime dysfunction. Responses and scored on a 0–3 ordinal scale, and the global score ranges between 0 and 21. A global score greater than 5 indicates poor sleep quality [47, 48].

The nine-item Patient Health Questionnaire (PHQ-9) gauges depressive symptom severity [49], while the seven-item Generalized Anxiety Disorder-7 (GAD-7) assesses generalized anxiety disorder symptoms [50]. Responses range between 0 (not at all) and 3 (nearly every day). Symptom severity is assessed as minimal (0–4), mild (5–9), moderate (10–14), or severe (≥ 15). Both instruments exhibited strong reliability and validity across diverse populations and settings [51].

The Perceived Stress Scale (PSS-10), a 10-item scale ranging from 0 (never) and 4 (very often), assesses subjective perceptions of stress. Total scores range between 0 and 40 [52]. The 12-item Multidimensional Scale of Perceived Social Support (MSPSS) assesses perceptions of social support [53]. Responses range between 1 to 7, where higher average scores signify higher perceived social support [54].

### Sociodemographic and lifestyle measurements

Sociodemographic data included age, gender, ethnicity, education, marital status, profession, housing type, personal monthly income, and residential area (S1 File), while lifestyle data included tobacco, alcohol, and caffeine consumption in the past 12 months. Physical activity levels were assessed by the validated and widely employed International Physical Activity Questionnaire (IPAQ) Short Form on physical activity across various domains, including leisure, work-related, and transportation-related activities, as well as household chores [55].

### Smartphone activities

Smartphone activities questions were adapted from a previous study [4] and included time spent on smartphones, and smartphone activities (S2 File). Smartphone time usage data was recoded into three groups: "< 2 hours," "2–4 hours," and "> 4 hours".

### Procedure

Household data collection was facilitated by a survey company in collaboration with the first author, who oversaw the training of data collectors. When available, validated translated instruments were employed; otherwise, the survey company utilized back translation [56] between English, Mandarin, and Malay. To boost response rates, notification letters informed residents in advance about the purpose and scheduling of the survey, and participants received a SGD$30 voucher. It took between 20 to 30 minutes to complete the survey, and data collectors made up to three attempts to reach households.

### Statistical analysis

#### Sample size calculation and sampling procedures

Sample size calculation was based on estimates by [34], based on which the Singapore Department of Statistics (SingStat) calculated an estimated sample size of 400, assuming prevalence of 39% at a 95% CI. To address non-response rates, we considered Singapore’s household survey figures, which reported rates of at least 50%, depending on wave [57, 58]. We therefore estimated a non-response rate of 65%, resulting in a rounded estimate of 1200 households to be surveyed. Households were selected based on a proportionate stratified random design by dwelling type (public flats, condominiums, and landed properties). From each selected household, one eligible individual was randomly chosen. The survey response rate was determined by dividing the number of completed surveys by the number of eligible cases (the individuals successfully identified at their households, excluding ineligible cases) and expressing this value as a percentage [59].

#### Analysis plan

Significant components identified in preliminary analyses were integrated into multivariable logistic regression models, adjusting for age, gender, ethnicity, and residential area. Odds ratio (OR) and 95% CIs were reported where appropriate. Given the absence of clinically validated cut-off scores for classifying PSU with the SAS-SV, a sensitivity analysis was conducted by treating SAS-SV scores as a continuous outcome, allowing examination of potential influences of SAS-SV cut-offs on observed effects. Exploratory, age-stratified, and gender-stratified analyses were performed to determine if the associated factors of PSU varied across age groups and gender. All analyses were conducted with SPSS software version 27.0 (IBM Corp. Armonk, NY, USA).

### Ethics

The study procedures were carried out in accordance with the Declaration of Helsinki. The SingHealth Centralised Institutional Review Board (CIRB Ref. No.: 2022/2123) approved the study. All participants were informed about the study, oral consent was obtained and documented in the study records, and a waiver of written informed consent documentation was granted by the ethics board based on ethical considerations.

## Results

### Participants characteristics

The overall survey response rate was 45.7%. Out of 875 eligible households, 400 successfully completed the survey. The characteristics of our sample is summarised in Table 1. The distribution of respondents’ age, gender, ethnicity, and marital status is similar to Singapore’s population census data (S1 Table).

**Table 1:** Profile of survey respondents.

Variables	n = 400
**Age, n (%)**	
21–30 years	105 (26.3)
31–40 years	87 (21.8)
41–50 years	92 (23.0)
51–60 years	116 (29.0)
**Education level, n (%)**	
Primary & below	25 (6.3)
Secondary / ‘O levels’	109 (27.3)
‘A’ levels / Diploma	119 (29.3)
University & above	147 (36.8)
**Gender, n (%)**	
Male	200 (50.0)
Female	200 (50.0)
**Marital status, n (%)**	
Single (never married, divorced, widowed)	163 (40.8)
Married	237 (59.3)
**Ethnicity, n (%)**	
Chinese	287 (71.8)
Malay	62 (15.5)
Indian	42 (10.5)
Others	9 (2.3)
**Profession, n (%)**	
Senior / Middle Management	55 (13.8)
Executive	77 (19.3)
Clerical	35 (8.8)
Blue Collar	63 (15.8)
Professionals (Doctor, lawyer etc.)	55 (13.8)
Others (Freelancers, Business owners, military servicemen)	27 (6.8)
Not working	88 (22.0)
**Personal monthly income, n (%)**	
Not working / $0	88 (22.0)
Below $2000	35 (8.8)
2000–3999	128 (32.0)
4000–5999	97 (24.3)
6000–9999	40 (10.0)
10000 & above	12 (3.0)
**Area of residence, n (%)**	
Bedok	152 (38.0)
Changi	10 (2.5)
Pasir Ris	51 (12.8)
Tampines	187 (46.8)
**Housing type, n (%)**	
1–2 room flats	18 (4.5)
3 room flats	97 (24.3)
4 room flats	169 (42.3)
5 room and executive	88 (22.0)
Condominiums or landed properties	28 (7.1)
**SAS-SV score mean (SD)**	27.8 (9.9)
**Sleep quality, n (%)**	
Good sleep quality	316 (79.0)
Poor sleep quality	84 (21.0)
**Mild to severe depressive symptoms, n (%)**	
No	333 (83.3)
Yes	67 (16.7)
**Mild to severe anxiety symptoms, n (%)**	
No	321 (80.3)
Yes	79 (19.7)
**MSPSS score mean (SD)**	5.53 (1.1)
**PSS-10 score mean (SD)**	15.8 (5.9)

**SAS-SV**: Smartphone Addiction Scale–Short Version; range 10 to 60; **Sleep quality**: Pittsburgh Sleep Quality Index; score of > 5 denotes poor sleep quality; **Depressive symptoms**: Patient Health Questionnaire-9; score of ≥ 5 denotes mild to severe symptoms; **Anxiety symptoms**: Generalised Anxiety Disorder-7; ≥ 5 denotes mild to severe symptoms; **MSPSS**: multidimensional scale of perceived support; range 1 to 7; **PSS-10**: Perceived Stress Scale; range 0 to 40.

The SAS-SV mean score was 27.8 (SD = 9.9). 136 individuals met the cut-off for PSU, indicating an estimated prevalence of 34.0%, (95% CI 29.4, 38.7). By gender, the prevalence of PSU was 29.5% in males, (95% CI 22.8 to 36.2) and 38.5% in females, (95% CI 31.8 to 45.2). Regarding age groups, the prevalence of PSU was 51.4% (n = 54) among respondents aged 21 through 30, (95% CI 41.3 to 61.6), 27.6% (n = 24) for 31 through 40, (95% CI 17.1 to 38.1), 33.7% (n = 31) for 41 through 50, (95% CI 22.5 to 44.9), and 23.3% (n = 27) for those aged 51 through 60, (95% CI 13.9 to 32.7).

### Associations of sociodemographic and lifestyle variables with PSU

Younger age and unmarried status were significantly associated with PSU (Table 2). Due to collinearity between marital status and age, marital status was excluded in subsequent multivariable logistic regression models. In the adjusted multivariable regression analyses (Table 3), significantly higher odds of PSU were observed for those aged 21 through 30 (OR = 3.72, 95% CI 2.06 to 6.74), *p* < 0.001) when compared to those aged 51 through 60.

**Table 2:** Variables associated with problematic smartphone use.

Variables	Total n (%)	PSU n (%)		p-value
		No (n = 264)	Yes (n = 136)	
**Age, n (%)**				
51–60 years	116 (29.0)	89 (33.7)	27 (19.9)	< 0.001*
41–50 years	92 (23.0)	61 (23.1)	31 (22.8)	
31–40 years	87 (21.8)	63 (23.9)	24 (17.6)	
21–30 years	105 (26.3)	51 (19.3)	54 (39.7)	
**Marital status, n (%)**				
Married	237 (59.3)	176 (66.7)	61 (44.9)	< 0.001*
Single (unmarried, widowed, divorced)	163 (38.7)	88 (33.3)	75 (55.1)	
**Sleep quality, n (%)**				
Good sleep quality	316 (79.0)	227 (86.0)	89 (65.4)	< 0.001*
Poor sleep quality	84 (21.0)	37 (14.0)	47 (34.6)	
**Mild to severe depressive symptoms, n (%)**				
No	333 (83.3)	235 (89.0)	98 (72.1)	< 0.001*
Yes	67 (16.7)	29 (11.0)	38 (27.9)	
**Mild to severe anxiety symptoms, n (%)**				
No	321 (80.3)	227 (86.0)	94 (69.1)	< 0.001*
Yes	79 (19.7)	37 (14.0)	42 (30.9)	
**Smartphone activities, n (%)**				
Calling family members	345 (86.3)	237 (89.8)	108 (79.4)	0.004*
Calling friends	335 (83.8)	228 (86.4)	107 (78.7)	0.048*
Using social media (Facebook etc.)	308 (77.0)	186 (70.5)	122 (89.7)	< 0.001*
Listening to music	240 (60.0)	144 (54.5)	96 (70.6)	0.002*
Watching videos / entertainment apps (Netflix etc.)	267 (66.8)	155 (58.7)	112 (82.4)	< 0.001*
**Smartphone usage time per day (past 30 days), n (%)**				
> 4 hours	120 (30.0)	57 (21.6)	63 (46.3)	< 0.001*
2 to 4 hours	178 (44.5)	127 (48.1)	51 (37.5)	
< 2 hours	102 (25.5)	80 (30.3)	22 (16.2)	
**MSPSS mean (SD)**	5.53 (1.1)	5.64 (0.93)	5.32 (1.32)	0.015*

**Sleep quality**: Pittsburgh Sleep Quality Index; score of > 5 denotes poor sleep quality; **Depressive symptoms**: Patient Health Questionnaire-9; score of ≥ 5 denotes mild to severe symptoms; **Anxiety symptoms**: Generalised Anxiety Disorder-7; ≥ 5 denotes mild to severe symptoms; **MSPSS**: multidimensional scale of perceived support; range 1 to 7.

**Table 3:** Adjusted multivariable logistic regression of factors associated with problematic smartphone use.

Variables	Multivariable adjusted OR (95% CI)	p-value
**Age**		
51–60 years	1.00	
41–50 years	1.69 (0.91–3.14)	0.098
31–40 years	1.33 (0.69–2.56)	0.387
21–30 years	3.72 (2.06–6.74)	< 0.001*
**Sleep quality**		
Good sleep quality	1.00	
Poor sleep quality	3.10 (1.85–5.21)	< 0.001*
**Mild to severe depression symptoms**		
No	1.00	
Yes	2.84 (1.61–5.02)	< 0.001*
**Mild to severe anxiety symptoms**		
No	1.00	
Yes	2.44 (1.44–4.15)	< 0.001*
**Smartphone activities**		
Calling family members	0.39 (0.21–0.73)	0.003*
Calling friends	0.53 (0.30–0.94)	0.030*
Using social media (Facebook, Instagram etc.)	2.81 (1.45–5.44)	0.002*
Listening to music	1.49 (0.92–2.42)	0.103
Watching videos / entertainment apps (e.g., Netflix)	2.76 (1.63–4.65)	< 0.001*
**Smartphone usage time per day (past 30 days)**		
> 4 hours	1.00	
2 to 4 hours	0.40 (0.24–0.66)	< 0.001*
< 2 hours	0.35 (0.18–0.67)	0.001*
**Higher levels of perceived social support**	0.76 (0.62–0.93)	0.007*

Logistic regression models adjusted for age, gender, ethnicity, and residential area

*Significant at the p < 0.05 level

### Associations of sleep quality and psychological well-being with PSU

Significant associations were observed between PSU and poor sleep quality, self-reported symptoms of depression, and anxiety. Higher social support scores were associated with non-PSU respondents (Table 2). In adjusted multivariable analyses (Table 3), respondents with depressive symptoms had significantly higher odds of PSU compared to those reporting no or minimal symptoms (OR = 2.84, 95% CI 1.61 to 5.02, *p* = 0.001). Similarly, respondents with anxiety symptoms exhibited significantly higher odds of PSU (OR = 2.44, 95% CI 1.44 to 4.15, *p* < 0.001). The analyses also revealed higher odds of PSU in respondents experiencing poor sleep quality (OR = 3.10, 95% CI 1.85 to 5.21, *p* < 0.001). In contrast, higher levels of perceived social support were negatively associated with PSU (OR = 0.76, 95% CI 0.62 to 0.93, *p* = 0.007).

### Associations of smartphone activities with PSU

Significant associations also exist between PSU and specific smartphone activities, including calling family or friends, accessing social media, listening to music, and video/entertainment apps, and the amount of time on smartphone (Table 2). Multivariable analyses revealed that social media users have significantly higher odds of PSU, compared to non-users (OR = 2.81, 95% CI 1.45 to 5.44, *p* = 0.002; Table 3), as does video/entertainment usage (OR = 2.76, 95% CI 1.63 to 4.65, *p* < 0.001). In contrast, respondents using smartphones to make calls to family or friends had lower odds of PSU, compared to non-callers (calling family: OR = 0.39, 95% CI 0.21 to 0.73, *p* = 0.003; calling friends: OR = 0.53, 95% CI 0.30 to 0.94, *p* = 0.030). Individuals spending four hours or less daily on smartphones exhibited lower odds of PSU, compared to those using smartphones for more than four hours (two to four hours: OR = 0.40, 95% CI 0.24 to 0.66, *p* < 0.001; less than two hours: OR = 0.35, 95% CI 0.18 to 0.67, *p* = 0.001).

A sensitivity analysis employing Smartphone Addiction Scale-Short Version (SAS-SV) as a continuous outcome variable yielded consistent findings with the primary analyses (S2 Table), which suggests that associations with PSU were not significantly influenced by the SAS-SV cut-off scores.

### Age-stratified analysis

Given the significant prevalence of PSU among individuals aged 21 through 30, age-stratified analyses were performed to compare this age group with those aged over 30 (Table 4). Adjusted multivariable logistic regression models in the 21–30 age bracket (n = 105) showed significant positive associations with PSU for being female (OR = 2.60, 95% CI 1.15 to 5.88, *p* = 0.022) and the use of smartphone video/entertainment apps (OR = 6.03, 95% CI 1.58 to 22.95, *p* < 0.003). In the aged 30 and above cohort (n = 295), significant associations with PSU included poor sleep quality (OR = 3.72, 95% CI 2.01 to 6.87, *p* < 0.001), depressive symptoms (OR = 3.83, 95% CI 1.95 to 7.49, *p* < 0.001), anxiety symptoms (OR = 2.59, 95% CI 1.36 to 4.90, *p* = 0.004), browsing social media (OR = 3.46, 95% CI 1.66 to 7.21, *p* < 0.001) or video/entertainment apps (OR = 2.23, 95% CI 1.27 to 3.94, *p* = 0.006). Conversely, smartphone use for calling family (OR = 0.41, 95% CI 0.20 to 0.83, *p* = 0.014) and friends (OR = 0.45, 95% CI 0.23 to 0.86, *p* = 0.015), using smartphones for four hours or less daily (two to four hours: OR = 0.22, 95% CI 0.11 to 0.43, *p*< 0.001; less than two hours: OR = 0.22, 95% CI 0.11 to 0.45, *p* < 0.001), as well as higher levels of perceived social support (OR = 0.64, 95% CI 0.50 to 0.81, *p* < 0.001) were negatively associated with PSU in participants aged 30 and above.

**Table 4:** Age-stratified adjusted multivariable logistic regression models.

	21–30 years old (n = 105)		> 30 years old (n = 295)	
Variables	Adjusted OR (95% CI)	p-value	Adjusted OR (95% CI)	p-value
**Gender**				
Male	1.00		1.00	
Female	2.60 (1.15–5.88)	0.022*	1.28 (0.76–2.17)	0.348
**Sleep quality**				
Good sleep quality	1.00		1.00	
Poor sleep quality	2.08 (0.79–5.48)	0.140	3.72 (2.01–6.87)	< 0.001*
**Mild to severe depressive symptoms**				
No	1.00		1.00	
Yes	1.74 (0.60–5.02)	0.306	3.83 (1.95–7.49)	< 0.001*
**Mild to severe anxiety symptoms**				
No	1.00		1.00	
Yes	2.69 (0.98–7.40)	0.055	2.59 (1.36–4.90)	0.004*
**Smartphone activities**				
Calling family members	0.38 (0.12–1.29)	0.122	0.41 (0.20–0.83)	0.014*
Calling friends	1.01 (0.31–3.29)	0.986	0.45 (0.23–0.86)	0.015*
Using social media	2.34 (0.40–13.71)	0.345	3.46 (1.66–7.21)	< 0.001*
Watching videos / entertainment apps	6.03 (1.58–22.95)	< 0.003*	2.23 (1.27–3.94)	0.006*
**Smartphone usage time per day (past 30 days)**				
> 4 hours	1.00		1.00	
2 to 4 hours	0.97 (0.42–2.25)	0.947	0.22 (0.11–0.43)	< 0.001*
< 2 hours	0.54 (0.08–3.66)	0.523	0.22 (0.11–0.45)	< 0.001*
**Higher levels of perceived social support**	0.99 (0.66–1.49)	0.973	0.64 (0.50–0.81)	< 0.001*

Logistic regression models adjusted for gender, ethnicity and residential area

*Significant at p < 0.05

### Gender-stratified analysis

Given the established evidence in the literature pointing to gender differences in PSU, we conducted an exploratory analysis to investigate this further. Our age-stratified analysis additionally revealed gender-based variations in PSU prevalence, supporting the association between gender and PSU. Gender-stratified logistic regression analyses were performed, with adjustments for age, ethnicity, and residential area (Table 5). Among males, those aged 21–30 years had significantly higher odds of PSU compared to those aged 51–60 years (OR = 3.27, 95% CI 1.30 to 8.22, *p* = 0.012). For females, individuals aged 21–30 years exhibited significantly higher odds of PSU (OR = 4.97, 95% CI 2.16 to 11.45, *p* < 0.001). Poor sleep quality was significantly associated with increased odds of PSU in both males (OR = 3.15, 95% CI 1.46 to 6.81, *p* = 0.003) and females (OR = 3.04, 95% CI 1.51 to 6.15, *p* = 0.002). Depressive symptoms were associated with higher odds of PSU for both genders, with an adjusted OR of 4.23, 95% CI [1.72, 10.44], *p* = 0.002 for males, and adjusted OR of 2.15, 95% CI [1.03, 4.49], *p* = 0.041 for females. Anxiety symptoms were also significantly associated with PSU, with an adjusted OR of 3.04, 95% CI [1.29, 7.15], *p* = 0.011 for males, and adjusted OR of 2.06, 95% CI [1.04, 4.10], *p* = 0.038 for females.

**Table 5:** Gender-stratified adjusted multivariable logistic regression models.

	Male (n = 200)		Female (n = 200)	
Variables	Adjusted OR (95% CI)	p-value	Adjusted OR (95% CI)	p-value
**Age**				
51–60 years	1.00		1.00	
41–50 years	1.69 (0.63–4.55)	0.297	1.88 (0.83–4.27)	0.129
31–40 years	2.49 (0.95–6.53)	0.064	0.65 (0.25–1.72)	0.389
21–30 years	3.27 (1.30–8.22)	0.012*	4.97 (2.16–11.45)	< 0.001*
**Sleep quality**				
Good sleep quality	1.00		1.00	
Poor sleep quality	3.15 (1.46–6.81)	0.003*	3.04 (1.51–6.15)	0.002*
**Mild to severe depressive symptoms**				
No	1.00		1.00	
Yes	4.23 (1.72–10.44)	0.002*	2.15 (1.03–4.49)	0.041*
**Mild to severe anxiety symptoms**				
No	1.00		1.00	
Yes	3.04 (1.29–7.15)	0.011*	2.06 (1.04–4.10)	0.038*
**Smartphone activities**				
Calling family members	0.49 (0.21–1.11)	0.088	0.42 (0.17–1.05)	0.064
Calling friends	0.37 (0.16–0.87)	0.022*	0.88 (0.41–1.89)	0.748
Using social media	2.77 (1.11–6.94)	0.030*	2.62 (1.03–6.67)	0.044*
Watching videos / entertainment apps	2.15 (1.03–4.49)	0.042*	3.91 (1.83–8.34)	< 0.001*
**Smartphone usage time per day (past 30 days)**				
> 4 hours	1.00		1.00	
2 to 4 hours	0.42 (0.20–0.89)	0.024*	0.37 (0.18–0.73)	0.004*
< 2 hours	0.44 (0.18–1.09)	0.077	0.27 (0.10–0.69)	0.007*
**Higher levels of perceived social support**	0.77 (0.58–1.02)	0.071	0.70 (0.52–0.94)	0.018*

Logistic regression models adjusted for age, ethnicity and residential area

*Significant at p < 0.05

Social media use was positively associated with PSU in males (OR = 2.77, 95% CI 1.11 to 6.94, p = 0.030) and females (OR = 2.62, 95% CI 1.03 to 6.67, *p* = 0.044). Watching videos or entertainment apps was also associated with higher odds of PSU in males (OR = 2.15, 95% CI 1.03 to 4.49, *p* = 0.042) and females (OR = 3.91, 95% CI 1.83 to 8.34, *p* < 0.001). Conversely, calling friends was associated with lower odds of PSU among males (OR = 0.37, 95% CI 0.16 to 0.87, *p* = 0.022) but not among females (*p* = 0.748). Smartphone usage of 2–4 hours per day was associated with lower odds of PSU relative to usage over 4 hours per day for males (OR = 0.42, 95% CI 0.20 to 0.89, *p* = 0.024) and females (OR = 0.37, 95% CI 0.18 to 0.73, *p* = 0.004). Among females, less than 2 hours of daily usage was also associated with significantly lower odds of PSU (OR = 0.27, 95% CI 0.10 to 0.69, *p* = 0.007). Higher perceived social support was associated with reduced odds of PSU in females (OR = 0.70, 95% CI 0.52 to 0.94, *p* = 0.018), although this association did not reach significance in males (*p* = 0.071).

## Discussion

The PSU prevalence of 34% among Singaporean adult residents surpasses the rates in Spain, Lebanon, and Germany [31–33], yet fall below Hong Kong and Tianjin [34, 60]. These variations may be due in part to cultural differences, as has also been proposed by [61] in a cross-cultural mixed methods study, where Chinese university students had significantly higher rates of PSU, compared to their British counterparts.

Our findings reveal a positive association between PSU and engagement with social media, in contrast to protective effects associated with social networking, such as phoning family and friends. This finding clearly points at the differential effects of smartphone use, depending on the nuanced ways individuals interact with technology. In this sense, positive and meaningful interactions with significant others via smartphones may either bypass or alleviate PSU. Research indeed indicates that messaging and digital connections, for example, are less likely associated with PSU [18].

Consistent with prior research among children and adolescents [11], our study reinforced the observation that younger individuals across gender in our diverse, multi-ethnic adult sample exhibited a heightened susceptibility to PSU, with results suggesting that females in the 21–30 age group displayed greater susceptibility compared to males. The observed age-related vulnerability may be linked to the type and degree of smartphone engagement among younger adults [62], which may render younger individuals more susceptible to gratification and reinforcement patterns and thus contribute to PSU [63]. Age-related difference could be associated with developmental and societal norms. The gender difference observed in the 21–30 age group is consistent with previous studies [64]. However, this gender difference was not present in the 30 and above group. The reason for this lack of gender difference in older age groups remains unclear and warrants further investigation.

Contrary to expectations and the reported 4.3% prevalence in the literature, [11], older individuals in our study exhibited a substantial PSU prevalence of 23%. This highlights the importance of intervention strategies that address diverse age groups, motivations, and needs. If PSU is viewed as stemming from underlying issues like loneliness and social isolation, interventions should target those specific needs. Looking beyond individual therapies like motivation enhancement and cognitive behavioural therapy [65], therapies that involve patients and their social setting to manage PSU may include family and couple therapy [66, 67]. Furthermore, practical psychosocial interventions can be beneficial, which may include participation in community activities, courses, group exercises, and support groups [68]. For elderly, especially less mobile patients or those experiencing age-related stressors such as caregiver burden or "empty nest” symptoms [69], community nursing or other care programs may effectively mitigate PSU [70].

Our findings also indicated that the associations between PSU, increased smartphone usage, and symptoms of poor mental well-being such as anxiety, depression, and sleep disturbances are also present in our adult sample among those aged 21 through 60, in both genders. Evidence suggests that excessive smartphone use, especially before bedtime, is linked to decreased sleep efficiency, poorer sleep quality, and delayed bedtime [71, 72]. Prospective longitudinal studies have also revealed the possibility of a bidirectional association between PSU, sleep quality and mental health symptoms, suggesting complex reciprocities [72–74]. The absence of a significant association between poor sleep quality and PSU among ages 21 to 30 is consistent with a prior study [75]. However, we observed a departure from this trend among participants older than 30, where significant associations were found between poor sleep quality, mental health symptoms, and PSU. The relationship between PSU, age-related mental health symptoms, and sleep quality is complex, and this relationship may be influenced by variations in psychological resilience, as resilience moderates the relationship between mental well-being and PSU [76]. It is also important to recognize the inherent limitation in our study, as sleep quality and mental health symptoms were assessed through self-reports.

While both genders demonstrated increased PSU risk associated with social media use and entertainment apps, gender-specific trends were observed in the types of smartphone activities linked to PSU. For instance, males had lower odds of PSU when frequently calling friends. This finding aligns with the observation that men may use smartphones differently than women, with potentially fewer social pressures tied to online engagement [20, 21]. Interestingly, when considering the role of social support, our findings indicate that higher levels of perceived social support was not protective against PSU among younger individuals (21–30 years). However, in individuals older than 30, greater perceived social support was protective against PSU. This suggests that the protective influence of social support might differ as social dynamics and support systems evolve with age. Additionally, higher perceived social support was significantly associated with reduced PSU odds in females but not males. The existing literature on gendered patterns of social connectivity offers a potential explanation [77]. Females are more likely to use social connectivity sites and apps, making smartphones a tool for maintaining and developing relationships. However, the presence of offline and external social support networks may reduce their reliance on smartphones for these purposes. In contrast, males may not experience the same protective benefit, due to differences in how they use smartphones or expect support from their social networks [78].

This study provides valuable insights into the prevalence of PSU, emphasizing its community-wide and age-sensitive significance among Singaporean adult residents. A notable strength lies in the inclusion of middle-aged adults within a multi-ethnic population, an aspect often overlooked in PSU research. Surveying a broad and diverse population enables a comprehensive examination of PSU across various age groups, contributing to a more inclusive understanding of PSU in the general population. The study’s sampling methodology resulted in a sample closely reflecting the general population, thus enhancing the external validity of the findings. However, some limitations need to be highlighted. Our sample is drawn from eastern Singaporean adult residents. Future research should include a wider geographic region and age groups to complement our understanding of PSU on a national level. The survey response rate of 45.7% suggests the possibility of respondent bias, although it aligns with reported response rates for household community surveys in Singapore [79]. The cross-sectional design limits the ability to establish temporal trends, cohort dynamics, and causal explanations. Longitudinal, even panel designs, possibly as part of a larger health programme, would provide stronger evidence regarding the temporal, age and cohort-specific, and causal nature of smartphone use and associated problematic behaviours. While the SAS-SV has demonstrated reliability and validity in previous studies [34, 45], the cut-off values used in this study have not been validated in the local population. Although sensitivity analyses were conducted to address this concern, interpreting the applicability of these cut-off values should be contextualised accordingly. Lastly, reliance on self-reported data introduces the possibility of recall bias or social desirability bias, potentially leading to measurement error.

## Conclusions

This study revealed that the prevalence of PSU among adults in Singapore is considerable and requires further research. Notably, younger age, mental health symptoms, poor sleep quality, and engagement with social media or video/entertainment apps were found to be associated with PSU. Conversely, higher levels of perceived social support, utilising smartphones for communication with family or friends, and lesser time spent on smartphones were negatively associated with PSU. Furthermore, the elevated rates of PSU among the older population, in contrast to other studies, gives cause for concern. These results highlight the need for more studies, including mixed methods designs, and the need for interventions and treatment programs. Evaluating and exploring effective interventions that foster genuine human connections, on and offline, may provide valuable insights for policymakers, and mental health professionals seeking to address the challenges posed by the digital era.

## Supporting information

S1 File Sociodemographics and lifestyle questionnaire. (DOCX)

S2 File Smartphone activities usage questions. (DOCX)

S1 Table Comparison of study sample profile with Singapore census data. (DOCX)

S2 Table Sensitivity analyses with Smartphone Addiction Scale–Short Version as a continuous outcome. (DOCX)
