# Supplementary material for: Prevalence and associations of problematic smartphone use with smartphone activities, psychological well-being, and sleep quality in a household survey of Singapore adults
Source: PLoS One. 2024 Dec 18;19(12):e0315364. doi: 10.1371/journal.pone.0315364 (PMC11654946; doi:10.1371/journal.pone.0315364)
Supplement: S1 File — (DOCX) [file pone.0315364.s001.docx]

S1 File - Sociodemographics and lifestyle questionnaire

**1. Have you smoked within the past 12 months?**

**Includes respondents who needed to stop smoking daily temporarily because of religious fasting or medical reasons*

| □ 1 | Daily |
| --- | --- |
| □ 2 | Occasionally |
| □ 3 | Stopped smoking completely |
| □ 4 | Have never smoked before |

**2. In the past 12 months, how frequently have you had at least one drink?**

**One standard alcoholic drink is defined as 1 can (330 mL) of regular beer, half glass (175 mL) of wine or 1 nip (35 mL) of spirit.*

| □ 1 | 5 or more days a week |
| --- | --- |
| □ 2 | 1 - 4 days per week |
| □ 3 | 3 days a month |
| □ 4 | No consumption of alcohol (proceed to Q4) |

**3. What is your main alcoholic drink?**

□ Beer

□ Stout

□ Wines (champagne, port)

□ Spirits (gin, whisky, rum, brandy, vodka)

□ Alcopops / other premixed drinks

□ Others (please specify: __________________________________________ ) □ No specific preference

**4. In the past 12 months, how frequently have you consumed these beverages?**

|  | | Never | 1-3 days a month | 1-4 days per week | 5 or more days a week |
| --- | --- | --- | --- | --- | --- |
| **a** | Coffee  ___ (237 ml) Standard cup |  |  |  |  |
| **b** | Decaf Coffee  ___ (237 ml) Standard cup |  |  |  |  |
| **c** | Tea  ___ (237 ml) Standard cup |  |  |  |  |
| **d** | Pepsi/Coke  ___ (355 ml) Standard can |  |  |  |  |
| **e** | Energy drink  ___ (355 ml) Standard can |  |  |  |  |

**5. What is your current age?**

_________________ (years old)

**6. What is your sex?**

□ 1) Male

□ 2) Female

**7. What is your race?**

□ 1) Chinese

□ 2) Malay

□ 3) Indian

□ 4) Others

**8. What is the highest level of education that you have attained?**

□ 1) No formal education / primary

□ 2) PSLE

□ 3) Secondary

□ 4) ‘O’ / ‘N’ level or NTC 3 certificate or its equivalent

□ 5) ‘A’ level or Polytechnic diploma

□ 6) University & above

**9. What is your current marital status?**

□ 1) Never married

□ 2) Married

□ 3) Divorced

□ 4) Widowed

**10. What is your main work status over the last 12 months?**

□ 1) Working → proceed to Q11

□ 2) Full-time student

□ 3) Homemaker or housewife

Proceed to Q13

□ 4) Retired

□ 5) Unemployed

**11. What is your occupation?**

□ 1) Senior Management (CEO, MD, GM, VP, Director, etc.)

□ 2) Middle Management (department manager, supervisory level)

□ 3) Executive

□ 4) Clerical (Clerks, Admin Assistant etc.)

□ 5) Blue-collar (Construction, Renovation, Hawker, Driver, Technician etc.)

□ 6) Professionals (Doctor, Lawyer, Architect, Consultant, Engineer, Lecturers etc.)

□ 7) Free-lancer (Tuition / Music Teacher, Artist, Designer, etc.)

□ 8) Sole Proprietor / Businessman

□ 9) Serving National Service-Including Regulars

□ 10) Others: _______________________

**12. What is your average personal monthly income over the last 12 months?**

□ 1) Below S$2,000

□ 2) S$2,000 – S$3,999

□ 3) S$4,000 – S$5,999

□ 4) S$6,000 – S$9,999

□ 5) S$10,000 – S$14,999

□ 6) S$15,000 & above

**13. How would you describe the place you live in?**

□ 1) HDB dwellings: 1- & 2-room flats □ 2) HDB dwellings: 3-room flats □ 3) HDB dwellings: 4-room flats □ 4) HDB dwellings: 5-room & executive flats □ 5) Condominiums & other apartments

□ 6) Landed properties

□ 7) Others, please specify __________________________________________

**14. What is your residential area?**

□ 1) Bedok

□ 2) Changi □ 3) Pasir Ris □ 4) Paya Lebar

□ 5) Tampines
