# Supplementary material for: Prevalence and associations of problematic smartphone use with smartphone activities, psychological well-being, and sleep quality in a household survey of Singapore adults
Source: PLoS One. 2024 Dec 18;19(12):e0315364. doi: 10.1371/journal.pone.0315364 (PMC11654946; doi:10.1371/journal.pone.0315364)
Supplement: S2 File — (DOCX) [file pone.0315364.s002.docx]

S2 File - Smartphone activities usage questions

**How many hours do you usually spend on your smartphone on average per day (the past 30 days)?**

- < 1 hr
- 1 to < 2 hr
- 2 to < 3 hr
- 3 to < 4 hr
- 4 to < 5 hr
- ≥ 5 hr

**Please select your smartphone usage activities (tick all that applies)**

- Calling family members
- Calling friends
- Texting (SMS, WhatsApp, WeChat etc.)
- Using the internet (google, reading the news etc.)
- Using social media (Facebook, Instagram etc.)
- Listening to music
- Playing games
- Watching videos / entertainment apps (e.g., Netflix)
- Work-related purposes (e.g., reply work messages or emails)
- Others (please specify): _____________________________
