# Supplementary material for: Prevalence and associations of problematic smartphone use with smartphone activities, psychological well-being, and sleep quality in a household survey of Singapore adults
Source: PLoS One. 2024 Dec 18;19(12):e0315364. doi: 10.1371/journal.pone.0315364 (PMC11654946; doi:10.1371/journal.pone.0315364)
Supplement: S1 Table — (DOCX) [file pone.0315364.s003.docx]

S3 Table - Comparison of study sample profile with Singapore census data

| Variables | Study Sample (2022) | Census Data (as at end-June 2022) * |
| --- | --- | --- |
| **Median age** | 42.0 | 42.1 |
| **Gender (%)** |  |  |
| Male | 50.0% | 48.9% |
| Female | 50.0% | 51.1% |
| **Marital status (%)** |  |  |
| Single | 40.8% | 40.0% |
| Married | 59.3% | 60.0% |
| **Education level (%)** |  |  |
| Primary & below | 6.3% | 21.6% |
| Secondary / ‘O levels’ | 27.3% | 16.5% |
| ‘A’ levels / Diploma | 29.3% | 25.7% |
| University & above | 36.8% | 36.1% |
| **Ethnicity (%)** |  |  |
| Chinese | 71.8% | 74.1% |
| Malay | 15.5% | 13.6% |
| Indian | 10.5% | 9.0% |
| Others | 2.3% | 3.3% |
| **Type of dwelling (%)** |  |  |
| Public flats | 93.0% | 78.6% |
| Condominiums & landed properties | 7.0% | 21.4% |

*Singapore Department of Statistics. (2022). Population Trends 2022. Retrieved May 2, 2024, from https://www.singstat.gov.sg/-/media/files/publications/population/population2022.ashx
