# Supplementary material for: Prevalence and associations of problematic smartphone use with smartphone activities, psychological well-being, and sleep quality in a household survey of Singapore adults
Source: PLoS One. 2024 Dec 18;19(12):e0315364. doi: 10.1371/journal.pone.0315364 (PMC11654946; doi:10.1371/journal.pone.0315364)
Supplement: S2 Table — (DOCX) [file pone.0315364.s004.docx]

S4 Table. Sensitivity analyses with Smartphone Addiction Scale – Short Version as a continuous outcome

| Variables | B  (95% CI)^ | Standard error | p-value |
| --- | --- | --- | --- |
| **Age** | -1.82 (-2.77 to -0.87) | 0.48 | < 0.001* |
| **Poor sleep quality** | 5.43 (3.15 to 7.72) | 1.16 | < 0.001* |
| **Depression** | 6.78 (4.27 to 9.29) | 1.28 | < 0.001* |
| **Anxiety** | 4.62 (2.24 to 7.00) | 1.21 | < 0.001* |
| **Smartphone activities** |  |  |  |
| Calling family members | -5.11 (-7.85 to -2.37) | 1.39 | < 0.001* |
| Calling friends | -1.67 (-2.95 to -0.39) | 0.65 | 0.011* |
| Using social media (Facebook, Instagram etc.) | 0.77 (0.29 to 1.25) | 0.24 | 0.002* |
| Listening to music | 0.33 (-0.02 to 0.67) | 0.18 | 0.062 |
| Watching videos / entertainment apps (e.g., Netflix) | 0.49 (0.24 to 0.74) | 0.13 | < 0.001* |
| **Lesser amount of time spent on smartphone per day (past 30 days)** | -4.12 (-0.88 to 0.12) | 0.67 | < 0.001* |
| **Higher levels of perceived social support** | -1.30 (-2.19 to -0.42) | 0.45 | 0.004* |

Linear regression models adjusted for age, gender ethnicity, education level, personal monthly income and residential area.

*Significant at the p < 0.05 level
